# Impact of IQ on the diagnostic yield of chromosomal microarray in a community sample of adults with schizophrenia

**DOI:** 10.1186/s13073-017-0488-z

**Published:** 2017-11-30

**Authors:** Chelsea Lowther, Daniele Merico, Gregory Costain, Jack Waserman, Kerry Boyd, Abdul Noor, Marsha Speevak, Dimitri J. Stavropoulos, John Wei, Anath C. Lionel, Christian R. Marshall, Stephen W. Scherer, Anne S. Bassett

**Affiliations:** 1 Clinical Genetics Research Program, Centre for Addiction and Mental Health, 33 Russell Street, Room 1100, Toronto, ON Canada M5S 2S1; 2 Institute of Medical Science, University of Toronto, Toronto, ON Canada; 3 Deep Genomics Inc, Toronto, ON Canada; 4 The Centre for Applied Genomics and Program in Genetics and Genome Biology, The Hospital for Sick Children, Toronto, ON Canada; 5 Division of Clinical and Metabolic Genetics, The Hospital for Sick Children, Toronto, ON Canada; 6 Humber River Regional Hospital, Toronto, ON Canada; 7 Department of Psychiatry & Behavioural Neurosciences, McMaster University, Hamilton, ON Canada; 8 Laboratory Medicine and Pathobiology, University of Toronto, Toronto, ON Canada; 9 Genome Diagnostics, Department of Paediatric Laboratory Medicine, The Hospital for Sick Children, Toronto, ON Canada; 10 Department of Molecular Genetics, University of Toronto, Toronto, ON Canada; 11 McLaughlin Centre, University of Toronto, Toronto, ON Canada; 12 Toronto General Research Institute, University Health Network, Toronto, ON Canada; 13 Cambell Family Mental Health Research Institute, Centre for Addiction and Mental Health, Toronto, ON Canada; 14 Department of Psychiatry, University of Toronto, Toronto, ON Canada

**Keywords:** Schizophrenia, Intellectual disability, IQ, Cognitive deficit, Copy number variation, Deletion, Duplication, Neurodevelopment, *ITPR1*, *SUMF1*

## Abstract

**Background:**

Schizophrenia is a severe psychiatric disorder associated with IQ deficits. Rare copy number variations (CNVs) have been established to play an important role in the etiology of schizophrenia. Several of the large rare CNVs associated with schizophrenia have been shown to negatively affect IQ in population-based controls where no major neuropsychiatric disorder is reported. The aim of this study was to examine the diagnostic yield of microarray testing and the functional impact of genome-wide rare CNVs in a community ascertained cohort of adults with schizophrenia and low (< 85) or average (≥ 85) IQ.

**Methods:**

We recruited 546 adults of European ancestry with schizophrenia from six community psychiatric clinics in Canada. Each individual was assigned to the low or average IQ group based on standardized tests and/or educational attainment. We used rigorous methods to detect genome-wide rare CNVs from high-resolution microarray data. We compared the burden of rare CNVs classified as pathogenic or as a variant of unknown significance (VUS) between each of the IQ groups and the genome-wide burden and functional impact of rare CNVs after excluding individuals with a pathogenic CNV.

**Results:**

There were 39/546 (7.1%; 95% confidence interval [CI] = 5.2–9.7%) schizophrenia participants with at least one pathogenic CNV detected, significantly more of whom were from the low IQ group (odds ratio [OR] = 5.01 [2.28–11.03], *p* = 0.0001). Secondary analyses revealed that individuals with schizophrenia and average IQ had the lowest yield of pathogenic CNVs (*n* = 9/325; 2.8%), followed by those with borderline intellectual functioning (*n* = 9/130; 6.9%), non-verbal learning disability (*n* = 6/29; 20.7%), and co-morbid intellectual disability (*n* = 15/62; 24.2%). There was no significant difference in the burden of rare CNVs classified as a VUS between any of the IQ subgroups. There was a significantly (*p*=0.002) increased burden of rare genic duplications in individuals with schizophrenia and low IQ that persisted after excluding individuals with a pathogenic CNV.

**Conclusions:**

Using high-resolution microarrays we were able to demonstrate for the first time that the burden of pathogenic CNVs in schizophrenia differs significantly between IQ subgroups. The results of this study have implications for clinical practice and may help inform future rare variant studies of schizophrenia using next-generation sequencing technologies.

**Electronic supplementary material:**

The online version of this article (doi:10.1186/s13073-017-0488-z) contains supplementary material, which is available to authorized users.

## Background

Schizophrenia is a severe psychiatric disorder associated with significant impairments in cognitive functioning [1]. On average, full scale IQ (FSIQ) is 7–8 points lower in cohorts with schizophrenia compared to general population norms [2] and the risk for schizophrenia has been shown to increase by 3.8% per 1-point decrease in FSIQ [3, 4]. However, this risk appears to be greatest for individuals with FSIQ < 85, and for those with a significantly lower performance IQ (PIQ) than verbal IQ (VIQ) (i.e. ~ 7-point difference or greater in the two main components of FSIQ) [4–6]. More extreme VIQ > PIQ discrepancies (i.e. ≥ 15 points) are clinically relevant and represent a neuropsychological hallmark of non-verbal learning disability (NVLD), a condition characterized by deficits in visual–spatial perception, complex psychomotor skills, non-verbal problem solving, arithmetic, and social judgment [7, 8]. The prevalence of schizophrenia in individuals with intellectual disability (ID; generally, IQ < 70) is threefold to fivefold higher than the general population prevalence of 1% [3, 9]. Taken together, these data suggest that the underlying genetic mechanisms that predispose individuals to schizophrenia may be stronger in those with low FSIQ, particularly low PIQ, than in those with higher IQ. Given that the IQ deficits in schizophrenia are associated with functional outcome [1], further study of genetic risk variants for schizophrenia in the context of the intellectual profile appears warranted.

Rare copy number variations (CNVs) have been identified to play an important role in the etiology of schizophrenia and developmental disability and/or ID (DD/ID) [10, 11]. Several large rare CNVs, including deletions at 2p16.3 overlapping *NRXN1*, 15q13.3 (BP4-BP5) deletions, and 16p11.2 deletions/duplications, have been identified in schizophrenia and DD/ID [12–14]. Additionally, CNVs associated with schizophrenia have been shown to negatively affect IQ in population-based controls without any major neuropsychiatric disorder [15]. The widespread use of clinical microarray testing in DD/ID has established the yield of pathogenic CNVs to be 15–20% [16]. In contrast, there have been significantly fewer diagnostic yield studies in schizophrenia [10, 17], possibly due to the lack of guidelines endorsing routine clinical microarray testing in this complex adult-onset condition [18]. Since most rare CNV studies of schizophrenia do not report IQ and/or have excluded participants with co-morbid ID [13, 19], the yield of pathogenic CNVs and the underlying genetic architecture of schizophrenia in the context of low IQ (schizophrenia-LIQ) remains unknown. Further, there have been no studies examining the genome-wide burden and/or functional impact of rare CNVs on schizophrenia while taking into account IQ, and after removing those CNVs that are deemed pathogenic.

Identifying sub-populations of individuals with schizophrenia who may be at an increased risk for a clinically reportable CNV, classified as pathogenic or a variant of unknown significance, would be useful for clinical practice. The primary aims of this study were twofold: (1) to compare the genome-wide burden of clinically reportable CNVs between individuals with schizophrenia-LIQ and schizophrenia-average IQ; and (2) to compare the genome-wide burden and functional impact of rare CNVs, beyond those that are currently deemed pathogenic, between individuals with schizophrenia-LIQ and average to higher IQ. Secondary analyses were aimed at identifying the yield of clinically reportable CNVs in schizophrenia across a wider range of IQ groups, including for those with a NVLD.

## Methods

### Schizophrenia sample collection and ascertainment

We recruited 688 adults who met the Diagnostic and Statistical Manual of Mental Disorders, Fourth Edition, diagnostic criteria for schizophrenia or schizoaffective disorder. Our detailed ascertainment strategy is described elsewhere [10]; however, it should be noted that the majority of the individuals recruited were chronically ill and therefore unlikely to include individuals in the first onset of illness whose diagnosis may change over time. There were 644 participants ascertained from six community mental health clinics across Central and Eastern Canada. In order to increase the number of individuals with schizophrenia at the lower end of the IQ spectrum we recruited an additional 44 participants with schizophrenia and ID from two outpatient mental health clinics that specialize in treating adults with a dual diagnosis (ID and a psychiatric disorder). However, of these 44 individuals, only 19 (43.2%) were included in the final cohort of 546 unrelated participants of European ancestry with adequate IQ data. The CNV data for a subset of the individuals with schizophrenia (*n* = 459; 66.7%) were previously published [10], although without the associated IQ data. Consent was obtained from all participants and surrogate consent was provided by an individual with power of attorney or equivalent for health decisions for individuals deemed incapable of providing informed consent. This study was approved by local institutional research ethics boards at the Centre for Addiction and Mental Health, Saint John Horizon Health Network, Humber River Hospital, Queen Elizabeth Hospital, Hamilton Health Services, and Bethesda Services.

### Clinical assessment of IQ level in individuals with schizophrenia

Similar to previous studies [20], we used a combination of previous IQ testing and educational attainment data to assign individuals with schizophrenia to an IQ subgroup. We also performed a comprehensive screening interview with each individual and/or his or her relative(s) to obtain medical, developmental, educational, and psychiatric history in addition to detailed demographic information. We retrospectively reviewed the available lifetime medical and psychiatric records for all 688 participants, blind to CNV status, and recorded results from all previous IQ and clinical genetic testing. These previous genetic and IQ results were not known at the time of recruitment. There were 212 of 546 (38.8%) individuals in the final sample with IQ scores (*n* = 136; 19.8%) and/or descriptive IQ ranges (*n* = 76; 11.0%) available (collectively referred to as IQ scores in the remaining text), 202 (36.9%) of whom had age at testing and schizophrenia age at onset both available. The majority of these IQ scores (n = 164/202; 81.2%) were obtained during the five years preceding the first onset of psychotic illness or within the 15 years after onset. Eighteen (8.9%) individuals had IQ testing completed more than five years before the first onset of psychotic illness and 20 (9.9%) had testing completed more than 15 years after onset. Individuals with IQ data had to be stable enough (e.g. with respect to psychotic symptoms) to be able to complete standardized IQ testing. There were no data on antipsychotic treatment at testing, but such treatment is unlikely to have affected IQ results [21].

We assigned individuals to the schizophrenia-LIQ or the schizophrenia-average IQ group if they had an IQ score of < 85 or ≥ 85, or an estimated IQ of borderline/ID or average range, respectively. For secondary analyses, individuals in the schizophrenia-LIQ group were divided into borderline intellectual functioning (IQ 71–85) or ID (IQ ≤ 70) groups. Given that the risk for schizophrenia may be higher for individuals with a significant discrepancy between their PIQ and VIQ scores we assigned individuals meeting criteria for a NVLD (PIQ ≥ 15 points lower than VIQ; Additional file 1: Figure S1) to a separate schizophrenia-NVLD category [6, 7]. We also used educational attainment to assign participants to intellectual functioning groups. However, IQ scores were deemed the more accurate measure of intellectual ability when years of education appeared out of keeping with expectations and functioning. Examples included individuals with IQ < 70 yet 12 years of education in a modified curriculum (assigned to schizophrenia-ID group) and individuals with IQ of 90 who left school to work after just eight years of education (assigned to schizophrenia-average IQ group).

In the absence of IQ scores we used educational attainment, which has a 0.6–0.7 correlation with FSIQ in the general population [22] and/or additional clinical data to assign individuals to each group as follows: the schizophrenia-LIQ group comprised individuals with a history of special education and/or had ID noted repeatedly throughout the medical records (estimated mild/moderate ID) and individuals who had 8–11 years of formal education with reported difficulties in school (e.g. repeated grades, enrolled in general courses in high school; estimated borderline intellectual functioning) [22, 23]. Years of education are not informative for individuals with schizophrenia-ID given that the majority of individuals are enrolled in special education and/or had modified academic curriculums. Individuals who had completed ≥ 12 years of education (graduated high school), had no reported difficulties in school, and had not repeated any grades were assigned to the schizophrenia-average IQ group [22, 23]. However, there were a number of scenarios in which our detailed clinical data led us to believe that an individual’s formal educational attainment did not reflect their true cognitive abilities. For example, we assigned individuals to the schizophrenia-average IQ group if they left school early due to incarceration, vocational and/or family responsibilities, or early onset of psychotic symptoms if they were reported to have done well academically up until that point. All assessments of IQ and educational attainment were performed blind to CNV status.

### CNV detection and annotation

High-quality genomic DNA was available for 540/546 (98.9%) participants and was submitted to the Centre for Applied Genomics in Toronto, Canada for genotyping using either the Affymetrix® Genome-Wide Human SNP array 6.0 or the CytoScan HD array. All samples met the Affymetrix quality control cut-offs. Similar to previous studies [10, 24], we only included CNVs that were > 10 kb, identified by at least two CNV calling algorithms (two of ChAS, iPattern, or Genotyping Console for the CytoScan HD array and two of iPattern, Birdsuite or Genotyping Console for the Affymetrix 6.0 array), spanning ten consecutive array probes, and overlapping < 75% of segmental duplications. Over 90% of CNVs called using these criteria validate using a second laboratory method [24]. The CytoScan HD array has a higher resolution than the Affymetrix 6.0 array; however, 90.0% of deletions ≥ 25 kb and spanning 25 consecutive array probes and duplications ≥ 50 kb and spanning 50 consecutive array probes are concordant between the two microarrays [25]. There was no significant difference in the proportion of individuals from the schizophrenia-LIQ or the schizophrenia-average IQ group analyzed on the Affymetrix 6.0 and CytoScanHD array (χ^2^ = 1.50, df = 1, *p* = 0.219). There were six (1.1% of 546) participants with 22q11.2 deletions included in the cohort who did not have Affymetrix 6.0 or CytoScan HD microarray data available and were therefore only included in the analyses comparing the burden of pathogenic CNVs.

We used 10,113 population-based controls (Additional file 1: Table S1) to adjudicate CNV rarity in the schizophrenia-LIQ and schizophrenia-average IQ groups. As before [10, 24, 26], we used a conservative definition of “rare,” defined as CNVs found in < 0.1% of these 10,113 independent controls using a 50% overlap criterion. Further quality control methods included removing locus-specific batch effects (i.e. CNVs with identical coordinates and copy number state that were present in > 1% of the sample) and manually joining large CNVs that appeared to be fragmented [13]. All CNV coordinates are given using the Genome Reference Consortium February 2009 build of the human genome (GRCh37/hg19).

### Assessment of ancestry and relatedness

We genotyped the 549,374 SNPs that are common to both the Affymetrix 6.0 and CytoScan HD arrays for participants using Birdseed v2 or Chromosomal Analysis Suite 3.1, respectively. Genotype data from 293,511 unlinked SNPs were used to estimate ancestry for the individuals with schizophrenia using PLINK [27]. Genotype data from 778 HapMap participants were used as a known reference for ancestry. Of the 688 individuals with schizophrenia in the original sample, 617 (89.6%) were identified to be of European descent. Pair-wise identity by descent analyses for individuals with high-resolution microarray data revealed that none of these participants were related to one another (all PI_HAT values were < 0.1). The unrelated individuals of European descent with schizophrenia who had sufficient IQ/educational data available to be categorized by intellect (*n*=546; 88.5% of 617) comprised the sample for this study.

### Clinical adjudication of rare CNVs in schizophrenia participants

All rare (< 0.1%) exonic CNVs > 100 kb and all non-coding CNVs > 500 kb were assessed for clinical relevance by a trained cytogeneticist following the American College of Medical Genetics (ACMG) guidelines for CNV interpretation [28]. CNVs were classified according to the five standard ACMG categories: (1) pathogenic; (2) variant of unknown significance (VUS) likely pathogenic; (3) VUS; (4) VUS likely benign; and (5) benign. We considered CNVs classified as pathogenic or VUS-likely pathogenic to be pathogenic. CNVs defined as clinically reportable included those classified as pathogenic, VUS-likely pathogenic, and VUS. The yield of pathogenic CNVs, VUS, and clinically reportable CNVs (pathogenic and VUS combined) were calculated based on the proportion of individuals in the schizophrenia-LIQ vs the schizophrenia-average IQ group with at least one of these CNV types, regardless of size or chromosomal location.

### Genome-wide CNV burden and statistical analyses

In our primary analyses, we tested the hypothesis that the genome-wide burden of clinically reportable CNVs was greater for participants with schizophrenia-LIQ than for those in the schizophrenia-average IQ group. In addition, after excluding individuals who were identified to have a pathogenic CNV (Table 1), which tend to be large and overlap many genes, we performed a logistic regression analysis to compare the total number, total length, and genic content of rare autosomal CNVs (all, deletions and duplications separately) > 10 kb between the schizophrenia-LIQ and schizophrenia-average IQ groups. Sex and genotyping platform were included as covariates. Odds ratios (ORs) and 95% confidence intervals (CIs) were calculated using R 3.3.1 software. All tests were two-sided with *p* < 0.05 defined for statistical significance, and uncorrected given limited multiple testing.

**Table 1:** Pathogenic CNVs (*n* = 41) identified in 39 unrelated participants with schizophrenia annotated by IQ group

No.	ID	IQ group	Sex	Chr	Cytoband	Start (hg 19)	Size (bp)	CN	Segdups^a^	Previously published^b^	Genes (n)	Selected candidate gene(s)^d^
1	2	Average	M	1	1q21.1	145,760,806	2,083,985	Gain	•	•	17	*BCL9*, *GJA5*, *PRKAB2*, *GJA8*, *PDZK1*
2	3	Average	F	1	1q21.1	145,932,468	1,898,716	Gain	•	•	15	*BCL9*, *GJA5*, *PRKAB2*, *GJA8*, *PDZK1*
3	562^f^	Mod ID	F	2	2p16.3	51,066,869	563,162	Loss		•^c^	1	*NRXN1*
4	570	BL	M	2	2p16.3	51,181,653	189,279	Loss		•^c^	1	*NRXN1*
5	7^f^	Average	M	2	2q13	111,388,632	1,727,361	Gain	•	•	9	*ANAPC1*, *BCL2L11*, *MERTK*
6	8	Average	M	2	2q13	111,388,632	1,727,361	Loss	•	•	9	*ANAPC1*, *BCL2L11*, *MERTK*
7	9	NVLD	M	2	2q13	111,388,632	1,727,361	Gain	•	•	9	*ANAPC1*, *BCL2L11*, *MERTK*
8	396	NVLD	M	3	3p26.1	4,418,429	277,309	Loss			3	*SUMF1*, *ITPR1*
9	13	NVLD	M	3	3q13.31	113,825,760	2,062,410	Loss		•	6	*LSAMP*, *DRD3*, *ZBTB20*, *GAP43*
10	452	Mild ID	M	3	3q27.1-q27.2	184,400,855	1,580,956	Loss			17	*TRA2B*
11	565^e,g^	BL	F	5	5p15.33-5p15.2	113,577	10,191,390	Loss			83	*IRX1*, *IRX2*, *IRX4*, *NDUFS6*, *SLC6A3*, *NSUN2*, *MTRR*, *CCT5*
12	17	Average	F	5	5p15.33-p15.32	1,811,574	3,687,431	Loss		•	7	*IRX1*, *IRX2*, *IRX4*, *NDUFS6*
13	247	Mild ID	F	6	6p25.3-p25.1	149,661	6,836,705	Loss		•	35	*FOXC1*, *GMDS*, *NRN1*, *TUBB2B*
14	565^e,g^	BL	F	6	6q26-q27	163,617,482	7,302,001	Gain			59	*RNASET2*, *TBP*
15	206	BL	F	7	7q22.2-q31.1	105,517,719	10,037,597	Loss		•	34	*COG5*, *DOCK4*, *FOXP2*, *GPR85*, *IMMP2L*, *LAMB1*, *NRCAM*, *PIK3CG*, *PNPLA8*
16	115	BL	F	8	8p23.3-p23.1	158,062	6,830,865	Loss		•	21	*ANGPT2*, *CLN8*, *CSMD1*, *DLGAP2*, *MCPH1*
17	40	Average	M	10	10q11.22-q11.23	46,485,761	5,173,684	Gain	•	•	42	*CHAT*, *ERCC6*, *GDF2*, *GPRIN2*, *MAPK8*, *SLC18A3*
18	569	Mild ID	M	13	13q14.13-q14.3	46,589,256	6,220,619	Loss	•		59	*HTRA2*, *SUCLA2*, *ITMB2*, *RB1*
19	48	BL	M	15	15q11.2-q13.1	20,181,700	6,498,447	Gain	•	•	121	*CYFIP1*, *GABRB3*, *GABRA5*, *GABRG3*, *MAGEL2*, *NDN*, *UBE3A*
20	556	Mild ID	M	15	15q11.2-q13.1	22,770,422	5,757,338	Gain	•		116	
21	427	NVLD	F	15	15q11.2-q13.1	23,290,799	5,353,780	Gain	•		115	*GABRB3*, *GABRA5*, *GABRG3*, *MAGEL2*, *NDN*, *UBE3A*
22	49	NVLD	M	15	15q11.2-q13.1	23,641,514	5,432,624	Gain	•	•	106	
23	50	Average	M	15	15q11.2-q13.1	23,641,514	4,892,894	Gain	•	•	101	
24	52^j^	Mod ID	F	15	15q13.2-q13.3	30,821,637	1,690,584	Loss	•	•	8	*CHRNA7*, *OTUD7A*, *TRPM1*
25	568	BL	M	16	16p11.2	29,432,213	744,308	Loss	•		40	*DOC2A*, *MAPK3*, *PRRT2*, *QPRT*, *SEZ6L2*, *TBX6*
26	55	Mild ID	F	16	16p11.2	29,567,309	624,599	Gain	•	•	26	
27	56^f^	Mild ID	F	16	16p11.2	29,567,309	659,635	Gain	•	•	33	
28	57^e^	Mild ID	M	16	16p11.2	29,567,309	624,599	Gain	•	•	26	
29	58	Mild ID	M	16	16p11.2	29,567,309	659,635	Gain	•	•	33	
30	277	Mild ID	F	19	19p13.3-p13.2	2,754,548	9,685,341	Gain		•	280	Various, including *DNMT1*, *DOCK6*
31	577	BL	M	22	22q11.2	18,890,046	2,831,545	Loss	•	•	46	Various, including *DGCR8*
32	581	Mild ID	F	22	22q11.2	18,890,046	2,831,545	Loss	•	•	46	
33	582	Mild ID	M	22	22q11.2	18,890,046	2,831,545	Loss	•	•	46	
34	579	BL	M	22	22q11.2	18,895,226	2,466,420	Loss	•	•	46	
35	580^i^	NVLD	F	22	22q11.2	18,916,840	1,395,833	Loss	•	•	29	
36	578^h^	Mild ID	F	22	22q11.2	20,717,655	1,087,074	Loss	•		16	*SNAP29*, *CRKL*
37	271	Average	F	X	X chr (46, XO)	-	155,065,370	Loss			829	Various, including *IL1RAPL1*, *SYN1*
38	173	BL	M	X	X chr (47, XXY)	-	155,065,370	Gain		•	829	
39	57^e^	Mild ID	M	X	X chr (47, XXY)	-	155,065,370	Gain		•	829	
40	194^f^	Average	M	X	Xp22.33-p22.2	2,400,835	11,075,950	Loss			40	*NLGN4X*, *VCX*, *MID1*
41	574^f^	Mild ID	F	X	Xp11.23-p11.22	48,178,414	4,508,892	Gain	•		97	Various, including *SHROOM4*, *WDR45*, *SYP*, *FTSJ1*

^a^Rare CNVs with one or both breakpoints falling within a segmental duplication are denoted by a bullet point

^b^All CNVs with a bullet point in this column were previously published in Costain et al. (2013) [10] or ^c^Lowther et al. (2017) [35]

^d^These candidate genes were previously reported in Costain et al. 2013 [10] or were based on a comparable method, i.e. genes associated with a neuropsychiatric or neurodevelopmental phenotype identified from a comprehensive literature search and/or in the Online Mendelian Inheritance in Man (http://www.omim.org/) database. Not every gene was systematically searched for those CNVs that overlapped ≥ 100 genes. Selected candidate genes are only reported once for recurrent CNVs

^e^These individuals carry a second CNV classified as pathogenic

^f^These individuals carry a second CNV classified as a VUS

^g^These two CNVs are part of an unbalanced translocation

^h,i^These are 22q11.2 deletions arising between low copy repeats: ^h^B-D (atypical deletion) and ^i^A-B (typical, short nested deletion), respectively

^j^Case 52 was identified to also have mosaic (6 of 24 cells) Turner syndrome by karyotype [10]

*ID* case identification number; *Chr* chromosome, *CN* copy number, *Segdups* flanking segmental duplication; *Genes (n)* number of RefSeq protein-coding genes overlapped, *Average* average IQ group, *BL* borderline IQ group, *ID* intellectual disability group (mild or moderate), *NVLD* non-verbal learning disability, *M* male, *F* female

### Gene-set enrichment analysis

We performed a gene-set enrichment analysis in order to determine if the functional impact of rare autosomal CNVs differed between the schizophrenia-LIQ and schizophrenia-average IQ groups. We tested 17 gene-sets that were postulated to play a role in the pathogenesis of schizophrenia and/or DD/ID. These included 15 sets that were significantly enriched for deletions (*n* = 15) or duplications (*n* = 1) in a recent large-scale CNV study of schizophrenia [13]. Briefly, these included two sets containing genes that are predicted to be targets of *FMR1* [29, 30], three sets containing genes coding for members of N-methyl-D-aspartate receptors (NMDAR), neuronal activity-regulated cytoskeleton-associated protein, and components of the postsynaptic density (PSD) [31], and ten sets associated with neuronal function, synaptic components, and/or neurological/neurodevelopmental phenotypes in humans (*n* = 7) or mice (*n* = 3) [13]. We also included two sets that comprised genes that were overlapped significantly more often by deletions (*n* = 1) or duplications (*n* = 1) in a clinically ascertained cohort with DD/ID compared to controls [12]. Detailed descriptions of how these 17 gene-sets were compiled are outlined in Additional file 2.

The gene-set enrichment analysis used a logistic regression deviance test [31] [R/Bioconductor package cnvGSA: Gene Set Analysis of (Rare) Copy Number Variants (version 1.18.0)] (https://www.bioconductor.org/packages/release/bioc/html/cnvGSA.html) to evaluate if the number of genes overlapped by rare exonic deletions or duplications in each individual for each of the gene-sets (i.e. gene-set specific exonic burden) was predictive of the participant being a member of the schizophrenia-LIQ or the schizophrenia-average IQ group. We included sex, genotyping platform, and the total number of genes overlapped by rare CNVs per individual as covariates. Multiple-testing correction (Benjamini–Hochberg false discovery rate [BH-FDR]) was performed separately for each gene-set and CNV type. Gene-sets with a BH-FDR < 10% and *p* value < 0.05 were considered to be significantly enriched [32, 33].

## Results

### Clinical features of the cohort

Of the 546 unrelated participants with schizophrenia of European descent, there were 325 (59.5%) assigned to the schizophrenia-average IQ group, 192 (35.2%) assigned to the schizophrenia-LIQ group, 130 (67.7%) with borderline intellectual functioning and 62 with mild (*n* = 57) or moderate (*n* = 5) ID, and 29 (5.3%) assigned to the schizophrenia-NVLD group. Total years of education were significantly lower (Mann–Whitney U = 6453.5, *p* < 0.0001) in the schizophrenia-borderline intellectual functioning group (median = 10; range = 5–16 years) compared to the schizophrenia-average IQ group (median = 12; range = 5–19 years) and not significantly different between the schizophrenia-average IQ and the schizophrenia-NVLD (median = 12; range = 7–18 years) groups (*p* = 0.385). Before involvement in this study, only seven (1.3%) individuals from the entire cohort had previously received clinical genetic testing, all of whom had been recruited from a specialized dual diagnosis clinic. These included six (9.6%) individuals from the schizophrenia-ID group and one (0.8%) individual from the schizophrenia-borderline intellectual functioning group. Further demographic and clinical data for the cohort are provided in Additional file 1: Table S2.

### Burden of pathogenic CNVs

**Fig. 1:**
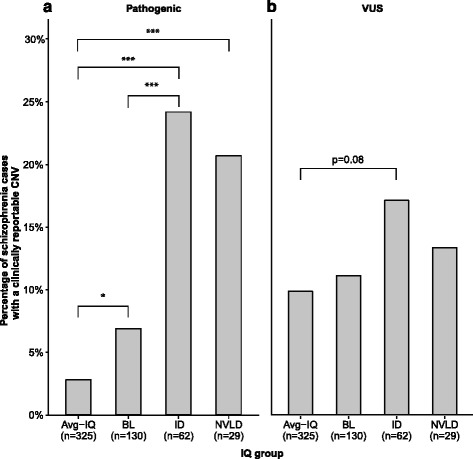
Yield of clinically reportable CNVs in schizophrenia by IQ group. The figure depicts the percentage of individuals with schizophrenia for each of the IQ groups with one or more pathogenic (defined as pathogenic or VUS-likely pathogenic) CNV (**a**) or one or more CNV classified as a VUS (**b**), determined using the ACMG guidelines for CNV interpretation [28]. Individuals with more than one clinically reportable CNV were only counted once. Schizophrenia participants were assigned to each of the IQ sub-groups using the methods described in the manuscript. *Average* average, *IQ* intelligence quotient, *BL* borderline intellectual functioning, *ID* intellectual disability, *NVLD* non-verbal learning disability, *VUS* variant of unknown significance. *Asterisks* above horizontal brackets represent the significance of the between-group comparisons: *p < 0.05, **p < 0.01, ***p < 0.001, NS = *p* > 0.1. All other comparisons were non-significant

There were 39/546 (7.1%; 95% CI = 5.2–9.7%) schizophrenia participants with at least one pathogenic CNV detected (Table 1). As hypothesized, our primary analysis revealed that the burden of pathogenic CNVs was higher in the schizophrenia-LIQ group (*n* = 24/192; 12.5%; 95% CI = 8.3–18.2%) compared to the schizophrenia-average IQ (*n* = 9/325; 2.8%; 95% CI = 1.3–5.3%) group (OR = 5.01 [2.28-11.03], p = 0.0001). There was no significant difference in the prevalence of pathogenic CNVs between the schizophrenia-ID and the schizophrenia-NVLD groups (*p* = 0.719) (Fig. 1). All six of the individuals with schizophrenia-NVLD and pathogenic CNV had a PIQ < 85 but only one had a VIQ < 85 (Additional file 1: Figure S1).

As part of secondary analyses, we divided the schizophrenia-LIQ cohort into subgroups (borderline intellectual functioning and ID), and determined that the majority of the schizophrenia-LIQ signal was coming from the schizophrenia-ID subgroup. There were 15/62 (24.2%; 95% CI 14.6-37.0%) participants with pathogenic CNVs in this subgroup compared to 9/130 (6.9%; 95% CI 3.4-13.1%) in the schizophrenia borderline intellectual functioning subgroup (Fig. 1). While the yield of pathogenic CNVs was significantly higher in those with borderline compared to those with average intellectual functioning (Fig. 1), this result became non-significant (*p* = 0.267) after removing the eight participants with borderline intellectual functioning who were recruited from a specialized dual diagnosis clinic. The overall pattern of results persisted when pathogenic deletions and duplications were analyzed separately (data not shown); however, the only comparisons that reached statistical significance were for an increased burden of pathogenic duplications in the schizophrenia-ID (*n* = 8; 12.9%) compared to the borderline intellectual functioning (*n* = 4; 3.1%; *p* = 0.015) or average IQ (*n* = 6; 1.8%; *p* = 0.0003) groups. There was no significant difference in the prevalence of pathogenic CNVs between the schizophrenia-ID and the schizophrenia-NVLD groups (*p* = 0.719) (Fig. 1).

There were 41 pathogenic CNVs (22 deletions, 19 duplications) with a median size of 2.83 Mb (range 189 kb to 155 Mb) identified in the 39 individuals (Table 1). Over half (25/41; 61.0%) of the CNVs had breakpoints that fell within segmental duplications. Many of these pathogenic CNVs have been previously associated with both schizophrenia and ID, including 1q21.1 duplications [34], 2p16.3 deletions overlapping *NRXN1* [35], 2q13 deletions/duplications [36, 37], 15q11.2-q13.1 duplications [38], typical 600 kb 16p11.2 deletion/duplications [13, 39], 22q11.2 deletions [40], and X chromosome abnormalities [17, 41]. There were also several CNVs at loci previously associated with ID but not schizophrenia, including a 3q27.1-q27.2 deletion [42], 6q26-q27 duplication [43], 13q14.13-q14.3 deletion [44], Xp11.23-p11.22 duplication [41], and Xp22.33-p22.2 deletion [45]. We also identified a novel 280 kb deletion at 3p26.1 overlapping schizophrenia candidate genes *ITPR1* [46] and *SUMF1* that has not been previously reported in the literature.

### Total burden of clinically reportable CNVs

There were 78 CNVs classified as VUS in 70 (12.8%; 95% CI 10.2-16.0%) schizophrenia participants (Additional file 3), five of whom also had a pathogenic CNV. In contrast to the pathogenic CNV results, there was no significant difference (*p* = 0.243) in the prevalence of individuals with one or more VUS between the schizophrenia-LIQ group (*n* = 26/192; 23.5%) and schizophrenia-average IQ group (*n* = 33/325; 10.2%). Secondary analyses revealed that there was also no significant difference in the prevalence of participants with a VUS between any of the IQ subgroups (Fig. 1). Of the 78 CNVs classified as VUS (median size 723 kb; range 115 kb to 4.3 Mb), there were slightly more duplications (*n* = 51; 65.3%) than deletions (*n* = 27; 34.7%), but this difference was non-significant (*p* = 0.057). Taken together, there were 99 (18.1%; 95% CI 15.0-21.7%) schizophrenia individuals with a clinically reportable CNV (pathogenic and/or VUS). Together there were 14 (2.6%) participants with two or more clinically reportable CNVs, significantly more of whom were in the schizophrenia-ID (*n* = 5/62; 8.1%) group compared to the schizophrenia-average IQ (*n* = 4/325; 1.2%) group (OR = 8.06 [2.21–32.93], *p* = 0.0018). There was no significant difference (*p* = 0.135) between the schizophrenia borderline intellectual functioning group (*n* = 5/130; 3.8%) and the schizophrenia-average IQ group.

### Burden of genome-wide rare CNVs

We also attempted to determine if the genome-wide burden of rare CNV was higher in an expanded schizophrenia-LIQ group compared to the schizophrenia-average IQ group, after excluding the 39 individuals with a pathogenic CNV. Given that the prevalence of pathogenic CNVs was similar for participants with ID and a NVLD, we added the 23 individuals with a NVLD (and no pathogenic CNV) to the original schizophrenia-LIQ group for the remaining analyses. After controlling for sex and genotyping platform there was no significant difference in the genome-wide burden, total genomic length, or total number of genes overlapped by rare autosomal CNVs between the two groups (Additional file 1: Table S3). However, there were significantly more genic CNVs in the expanded schizophrenia-LIQ group compared to the schizophrenia-average IQ group (OR = 1.19 [1.01–1.41], *p* = 0.042), primarily driven by an increased burden of genic duplications (OR = 1.42 [1.14–1.81], *p* = 0.002); findings for genic deletions did not reach significance (*p* = 0.129) (Additional file 1: Table S3).

### Gene-set enrichment analysis

After multiple-test correction (BH-FDR < 10% and *p* value < 0.05), we detected no gene-sets that were significantly enriched for rare autosomal deletions in the expanded schizophrenia-LIQ group (comprising individuals with schizophrenia and ID, borderline intellectual functioning, or NVLD) compared to the schizophrenia-average IQ group (data not shown). There was one gene-set, GO nervous system development, that was significantly (*p* = 0.013) enriched for rare duplications in the schizophrenia-LIQ group that had a BH-FDR of 0.22 (Table 2). To see if we could improve the FDR, we reduced the 17 gene-sets to only those six reported to have a FDR < 30% for rare genic duplications in the recent Psychiatric Genomics Consortium schizophrenia case control CNV study [13]. This resulted in an improved FDR (from 0.22 to 0.07) for the GO nervous system development gene-set. The GO nervous system development gene-set became non-significant (*p* = 0.074, FDR = 0.37) after excluding *n* = 39 participants with an pathogenic CNV (Table 2).

**Table 2:** Six gene-sets showing enrichment for rare duplications in the schizophrenia-LIQ group compared to the schizophrenia-average IQ group

		Schizophrenia-LIQ (n = 162)^a^			Schizophrenia-average IQ (n = 278)^a^			Analyses		
			Participants			Participants				
Gene-set name	Total genes (n)	CNVs (n)	n	%	CNVs (n)	n	%	*p*	BH-FDR	OR
GO Nervous system development	1874	44	35	18.5	29	28	10.1	0.013	0.0783	1.7
Union inclusive	2874	56	45	23.8	43	41	14.7	0.058	0.1554	1.4
NMDAR^b^	62	4	4	2.1	0	0	0	0.078	0.1554	Inf
Targets of *FMR1*, Darnell et al. (2012)	840	25	24	12.7	21	20	7.2	0.371	0.5563	1.3
GO Synaptic	622	15	15	8.5	11	11	4.0	0.471	0.5650	1.2
GO Nervous system transmission	716	18	17	9.0	10	10	3.6	0.761	0.7605	1.1

^a^All rare (< 0.1%) autosomal CNVs > 10 kb were included in the analysis; see “Methods” for details. Sample sizes represent those individuals with one or more rare CNVs overlapping at least 1 bp of coding sequence, according to RefSeq annotations, in each group (162/192, or 84.3%, of the schizophrenia-LIQ group; 278/325, or 85.5%, of the schizophrenia-average IQ group)

^b^The CNVs comprised four pathogenic 16p11.2 duplications in four individuals, for which the contributing gene for this gene-set result was *MAPK3*

*LIQ* low IQ, *GO* gene ontology, *CNV* copy number variation, *p* statistical result when all rare autosomal CNVs are included; *BH-FDR* Benjamini–Hochberg false discovery rate, *Inf* infinity, *NMDAR* N-methyl-D-aspartate receptor components

There were 44 rare duplications in 35 individuals in the expanded schizophrenia-LIQ group and 29 rare duplications in 28 individuals in the schizophrenia-average IQ group contributing to the GO nervous system development gene-set result (Additional file 3). The duplications not currently classified as pathogenic or a VUS in the schizophrenia-LIQ individuals overlapped several interesting neuropsychiatric candidate genes, such as *CNTN4*, *NDUFV2*, and *RCAN1* [47–49]. There were also duplications in two participants from the expanded schizophrenia-LIQ group that overlapped two genes (*ARSA* and *EIF2B1*) associated with leukodystrophy, a progressive disease that causes abnormal development of and/or destruction of the myelin sheath and can present in adulthood with symptoms similar to that of schizophrenia [50, 51].

## Discussion

This is the first study to examine the burden of clinically reportable CNVs in schizophrenia by IQ group. Our results revealed that 7.1% of schizophrenia individuals ascertained from a community outpatient setting may have a pathogenic CNV detected by genome-wide microarray. However, this diagnostic yield was not uniformly distributed across the cohort as there was a significant increase in the yield of pathogenic CNVs as IQ decreased (Fig. 1). We also demonstrated for the first time that the prevalence of pathogenic CNVs may be similar for individuals with schizophrenia-ID and schizophrenia-NVLD. Further, we identified an increased burden of rare genic autosomal duplications in the schizophrenia-LIQ compared to the schizophrenia-average IQ group, a finding that was not attributable to large rare pathogenic CNVs.

### The importance of clinical microarray testing in the dual diagnosis adult population

In the current study, the highest yield of pathogenic CNVs (24.1%) was identified in individuals with schizophrenia and co-morbid ID. This yield was higher than that that reported for epilepsy (~ 5–10%) [52] or ASD (~ 10–15%) [53] alone, and comparable to that for DD/ID (~ 15–20%) [16]. There have been few studies that have examined the burden of pathogenic CNVs in adults with a dual diagnosis (ID plus one or more additional neurodevelopmental and/or neuropsychiatric conditions), and even fewer that have focused specifically on schizophrenia-ID. A recent study identified a pathogenic CNV in nine of 72 (12.5%) individuals with ID and psychosis [54], about half of the yield reported in the current study. Such discrepancies in the diagnostic yield may be due to differences in ID severity between these two cohorts (data unavailable for the Wolfe et al. cohort) [54] and/or differences in the application of guidelines for CNV interpretation that are vulnerable to subjective evaluations of the evidence supporting the classification of a variant [55]. Further studies examining the diagnostic yield of microarray in the schizophrenia-ID population will be needed to clarify this.

With respect to other neuropsychiatric conditions, the rate of rare de novo CNVs has been shown to be increase with decreasing IQ individuals with ASD [56] but no studies have yet formally examined the diagnostic yield of clinical microarray in the ASD-ID population. Conversely, a recent study investigating adults with ID and pediatric-onset epilepsy revealed that 16.0% (*n* = 23/143) of the cohort had a pathogenic CNV [57], a higher prevalence than that reported for just epilepsy [52]. Taken together, these data suggest that adults with a dual diagnosis should be prioritized for clinical microarray testing. However, it is important to note that before inclusion in our study fewer than 10% of the dual diagnosis schizophrenia-ID participants in our cohort had received any type of clinical genetic testing despite meeting criteria for routine CMA testing based on the Miller et al. 2010 clinical recommendations [16]. This suggests that additional efforts to increase widespread CMA testing in the adult DD/ID population, particularly for those with a dual diagnosis, are needed.

### Increased burden of clinically relevant CNVs in individuals with schizophrenia-NVLD

To our knowledge, this is the first study to report on the burden of clinically reportable CNVs in individuals with schizophrenia-NVLD. Individuals with a NVLD demonstrate significant deficits in visual–spatial organization, motor coordination, and social perception and interaction, but retain relatively well-developed verbal skills [7]. Determining a diagnosis of NVLD relies heavily on formal IQ testing, allowing for the detection of significant VIQ > PIQ discrepancies that are difficult to ascertain clinically. Perhaps unsurprisingly, given the emphasis on verbal skills in formal education, there was no significant difference in the total years of education between the schizophrenia-NVLD and schizophrenia-average IQ groups (Additional file 1: Table S2). Yet, the schizophrenia-NVLD participants had a significantly higher burden of pathogenic CNVs compared to the schizophrenia-average IQ group. Indeed, the burden of pathogenic CNVs in the schizophrenia-NVLD group was more comparable to that of the schizophrenia-ID group (20.7% vs 24.1%). Interestingly, all of the individuals with schizophrenia-NVLD and a pathogenic CNV had a PIQ < 85, yet VIQ for all but one individual was in the average to above average range (Additional file 1: Figure S1). This finding has important clinical relevance because individuals with schizophrenia-NVLD would not generally be considered for clinical microarray testing [16].

NVLD has been previously described as being associated with several structural variants, including those underlying Turner syndrome [58], Williams syndrome [59], and the 22q11.2 deletion syndrome [60]. This study extends these findings to include other rare recurrent CNVs, including 15q11.2-q13.1 and 16p11.3 duplications. We also identified a novel 280-kb deletion at 3p26.1 overlapping genes *SUMF1* and *ITPR1* in a participant with schizophrenia-NVLD that has not been previously reported in the literature. *ITPR1* encodes the inositol 1,4,5-triphosphate receptor that plays an important role in releasing Ca^2+^ from the endoplasmic reticulum [61]. Interestingly, a recent whole-exome sequencing (WES) study identified 11 individuals with schizophrenia who had ultra-rare disrupting/damaging variants in *ITPR1* [46]. There is also a 60-kb deletion overlapping the first four exons of *ITPR1* in a case (nsv996226) with autistic behavior reported in the Clinical Genome Resource database (https://www.clinicalgenome.org). Heterozygous deletions and missense mutations in this gene have also been associated with adult onset spinocerebellar ataxia-15 (MIM 606658) and childhood onset spinocerebellar ataxia-29 (MIM 117360) [62, 63]. Additionally, homozygous/compound heterozygous truncating mutations and heterozygous deletions and missense mutations in *ITPR1* have been associated with Gillespie syndrome (MIM 206700), a disorder characterized by hypotonia, progressive hypoplasia, ataxia, and variable cognitive impairment with onset occurring within the first year of life [61]. Notably, many genes associated with spinocerebellar ataxia are also reported to play a possible role in schizophrenia, such as *ATAXN1*, *ATAXN2*, and *ATAXN10* [64–66].

### Delineating the genetic architecture of schizophrenia

Data from this study demonstrate that the genetic architecture of schizophrenia-LIQ, and probably schizophrenia-NVLD, differs significantly from schizophrenia-average IQ, even after excluding large rare pathogenic CNVs that have a well-documented impact on cognition in the general population [15]. Despite having a relatively small sample size, we were able to identify an increased burden of rare exonic duplications in the schizophrenia-LIQ group that may overlap more genes involved in nervous system development (Table 2). Larger sample sizes could help provide improved statistical support for these findings as well as potentially identify additional pathways relevant to schizophrenia-LIQ. Data from a recent WES study in schizophrenia identified a significantly increased burden of rare damaging variants in loss-of-function (LoF) intolerant genes associated with developmental disorders in individuals with schizophrenia-ID compared to those with schizophrenia and average IQ [20]. Interestingly, the burden of these damaging variants in LoF intolerant genes was also increased in schizophrenia-average IQ compared to controls, suggesting that they contribute to risk for developing schizophrenia but to a lesser degree than that for schizophrenia-ID [20].

### Advantages and limitations

Significant strengths of our study included the robust CNV detection methods used and systematic application of established guidelines for CNV interpretation [28]. Also, the community-based ascertainment strategy more closely reflects the general schizophrenia population than a strictly hospital-based recruitment strategy, thus allowing for more generalizable diagnostic yield estimates. The comprehensive phenotyping protocol facilitated our ability to stratify the schizophrenia cohort by IQ. Yet, despite substantial efforts, the numbers of individuals with schizophrenia-ID remained relatively small. We therefore targeted specialized dual diagnosis clinics in order to increase recruitment at the lower end of the IQ range. This appeared to increase the yield of pathogenic CNVs detected in the borderline intellectual functioning group, possibly reflecting an ascertainment bias in which more severely affected and/or psychiatrically complex individuals may be referred to such specialized clinics. Efforts to identify more individuals from community settings are needed.

Another limitation of our study was that although there were formal IQ data for a substantial number of individuals, educational data were used to group the remainder of the cohort. While the correlation between FSIQ and educational attainment is high (0.6–0.7) [22], it is possible that we have misclassified some participants, particularly those with a learning disability (such as NVLD) that cannot be delineated by educational attainment alone. Further, given that there are some data to suggest that there is an IQ decline associated with schizophrenia illness onset [67] and that the majority of the IQ scores used in this study were obtained just before or sometime after the onset of illness, it is possible that some individuals were incorrectly assigned to an IQ group lower than their “premorbid” IQ score would have placed them. However, we based IQ group placement on multiple pieces of evidence, including IQ scores, educational attainment, functioning, and personal circumstances. This process mirrors the multiple factors taken into consideration when making a clinical diagnosis of intellectual functioning. Also, each IQ group covers a fairly wide range of IQ scores. We therefore believe that IQ group misclassification is likely to be low for this study.

## Conclusions

Using high-resolution microarrays, the results indicate that the burden of pathogenic CNVs is significantly greater for individuals with schizophrenia and low IQ compared to those with normal to superior IQ. These data have important clinical and research implications, including demonstrating that participants with schizophrenia and low IQ should be prioritized for clinical microarray testing and highlighting the importance of taking IQ into account for the interpretation of future rare variant studies of schizophrenia. The data also suggest that individuals with schizophrenia-NVLD, comprising 5.3% of the sample, may also have an increased burden of pathogenic CNVs. Data from next-generation sequencing will allow for the detection of sequence variants and smaller structural variants that may help shed light on more specific mechanisms related to schizophrenia-LIQ. While we identified several potential candidate genes for schizophrenia-LIQ, larger samples will be required to provide sufficient statistical support for any given loci.

## Additional files

Additional file 1: A word document that contains one figure that depicts the verbal and performance IQ scores for 29 individuals with schizophrenia and a NVLD (**Figure S1**), and three tables, including: (1) a list of 10,113 population-based controls used to adjudicate CNV rarity in schizophrenia participants (**Table S1**); (2) the demographic and clinical information for 546 probands with schizophrenia of European ancestry (**Table S2**); and (3) the genome-wide burden of all rare autosomal CNVs > 10 kb between the expanded schizophrenia-LIQ and schizophrenia-average IQ groups (**Table S3**). (DOCX 87 kb)

Additional file 2: Description of the 17 gene-sets used in the gene-set enrichment analysis. (XLSX 13 kb)

Additional file 3: All rare (< 0.1% in population controls) CNVs used for the analyses in this study. (XLSX 199 kb)

## Abbreviations

ASD: Autism spectrum disorder
BH-FDR: Benjamini–Hochberg false discovery rate
CNV: Copy number variation
DD: Developmental delay
FSIQ: Full scale IQ
ID: Intellectual disability
IQ: Intelligence quotient
LIQ: Low IQ
LoF: Loss of function
NMDAR: N-methyl-D-aspartate receptor
NVLD: Non-verbal learning disability
OR: Odds ratio
PIQ: Performance IQ
VIQ: Verbal IQ
VUS: Variant of unknown significance

## References

[1] Kahn RS, Keefe RS (2013). Schizophrenia is a cognitive illness: time for a change in focus. JAMA Psychiatry..

[2] Woodberry KA, Giuliano AJ, Seidman LJ (2008). Premorbid IQ in schizophrenia: a meta-analytic review. Am J Psychiatry..

[3] Khandaker GM, Barnett JH, White IR, Jones PB (2011). A quantitative meta-analysis of population-based studies of premorbid intelligence and schizophrenia. Schizophr Res..

[4] Kendler KS, Ohlsson H, Sundquist J, Sundquist K (2015). IQ and schizophrenia in a Swedish national sample: their causal relationship and the interaction of IQ with genetic risk. Am J Psychiatry..

[5] Schulz J, Sundin J, Leask S, Done DJ (2014). Risk of adult schizophrenia and its relationship to childhood IQ in the 1958 British birth cohort. Schizophr Bull..

[6] Amminger GP, Schlogelhofer M, Lehner T, Looser Ott S, Friedrich MH, Aschauer HN (2000). Premorbid performance IQ deficit in schizophrenia. Acta Psychiatr Scand..

[7] Harnadek MC, Rourke BP (1994). Principal identifying features of the syndrome of nonverbal learning disabilities in children. J Learn Disabil..

[8] Purcell DW, Lewine RR, Caudle J, Price LR (1998). Sex differences in verbal IQ-performance IQ discrepancies among patients with schizophrenia and normal volunteers. J Abnorm Psychol..

[9] Morgan VA, Leonard H, Bourke J, Jablensky A (2008). Intellectual disability co-occurring with schizophrenia and other psychiatric illness: population-based study. Br J Psychiatry..

[10] Costain G, Lionel A, Merico D, Forsythe P, Russell K, Lowther C (2013). Pathogenic rare copy number variants in community-based schizophrenia suggest a potential role for clinical microarrays. Hum Mol Genet..

[11] Cooper GM, Coe BP, Girirajan S, Rosenfeld JA, Vu TH, Baker C (2011). A copy number variation morbidity map of developmental delay. Nat Genet..

[12] Coe BP, Witherspoon K, Rosenfeld JA, van Bon BW, Vulto-van Silfhout AT, Bosco P (2014). Refining analyses of copy number variation identifies specific genes associated with developmental delay. Nat Genet..

[13] Marshall CR, Howrigan DP, Merico D, Thiruvahindrapuram B, Wu W, Greer DS (2017). Contribution of copy number variants to schizophrenia from a genome-wide study of 41,321 subjects. Nat Genet..

[14] Rees E, Walters JT, Georgieva L, Isles AR, Chambert KD, Richards AL (2014). Analysis of copy number variations at 15 schizophrenia-associated loci. Br J Psychiatry..

[15] Stefansson H, Meyer-Lindenberg A, Steinberg S, Magnusdottir B, Morgen K, Arnarsdottir S (2014). CNVs conferring risk of autism or schizophrenia affect cognition in controls. Nature..

[16] Miller DT, Adam MP, Aradhya S, Biesecker LG, Brothman AR, Carter NP (2010). Consensus statement: chromosomal microarray is a first-tier clinical diagnostic test for individuals with developmental disabilities or congenital anomalies. Am J Hum Genet..

[17] Kushima I, Aleksic B, Nakatochi M, Shimamura T, Shiino T, Yoshimi A (2017). High-resolution copy number variation analysis of schizophrenia in Japan. Mol Psychiatry..

[18] Baker K, Costain G, Fung WLA, Bassett AS (2014). Chromosomal microarray analysis - a routine clinical genetic test for patients with schizophrenia. Lancet Psychiatry..

[19] International Schizophrenia Consortium (2008). Rare chromosomal deletions and duplications increase risk of schizophrenia. Nature.

[20] Singh T, Walters JTR, Johnstone M, Curtis D, Suvisaari J, Torniainen M (2017). The contribution of rare variants to risk of schizophrenia in individuals with and without intellectual disability. Nat Genet..

[21] Keefe RS, Bilder RM, Davis SM, Harvey PD, Palmer BW, Gold JM (2007). Neurocognitive effects of antipsychotic medications in patients with chronic schizophrenia in the CATIE Trial. Arch Gen Psychiatry..

[22] Kaufman AS, Kaufman JC, Liu X, Johnson CK (2009). How do educational attainment and gender relate to fluid intelligence, crystallized intelligence, and academic skills at ages 22-90 years?. Arch Clin Neuropsychol..

[23] Matarazzo JD, Herman DO (1984). Relationship of education and IQ in the WAIS—R standardization sample. J Consult Clin Psychol..

[24] Lionel AC, Crosbie J, Barbosa N, Goodale T, Thiruvahindrapuram B, Rickaby J (2011). Rare copy number variation discovery and cross-disorder comparisons identify risk genes for ADHD. Sci Transl Med.

[25] Uddin M, Thiruvahindrapuram B, Walker S, Wang Z, Hu P, Lamoureux S (2015). A high-resolution copy-number variation resource for clinical and population genetics. Genet Med..

[26] Silversides CK, Lionel AC, Costain G, Merico D, Migita O, Liu B (2012). Rare copy number variations in adults with tetralogy of Fallot implicate novel risk gene pathways. PLoS Genet..

[27] Purcell S, Neale B, Todd-Brown K, Thomas L, Ferreira MA, Bender D (2007). PLINK: a tool set for whole-genome association and population-based linkage analyses. Am J Hum Genet..

[28] Kearney HM, Thorland EC, Brown KK, Quintero-Rivera F, South ST, Working Group of the American College of Medical Genetics Laboratory Quality Assurance Committee (2011). American College of Medical Genetics standards and guidelines for interpretation and reporting of postnatal constitutional copy number variants. Genet Med.

[29] Darnell JC, Van Driesche SJ, Zhang C, Hung KY, Mele A, Fraser CE (2011). FMRP stalls ribosomal translocation on mRNAs linked to synaptic function and autism. Cell..

[30] Ascano M, Mukherjee N, Bandaru P, Miller JB, Nusbaum JD, Corcoran DL (2012). FMRP targets distinct mRNA sequence elements to regulate protein expression. Nature..

[31] Kirov G, Pocklington AJ, Holmans P, Ivanov D, Ikeda M, Ruderfer D (2012). De novo CNV analysis implicates specific abnormalities of postsynaptic signalling complexes in the pathogenesis of schizophrenia. Mol Psychiatry..

[32] Merico D, Zarrei M, Costain G, Ogura L, Alipanahi B, Gazzellone MJ (2015). Whole-genome sequencing suggests schizophrenia risk mechanisms in humans with 22q11.2 deletion syndrome. G3 (Bethesda).

[33] Stark KL, Xu B, Bagchi A, Lai WS, Liu H, Hsu R (2008). Altered brain microRNA biogenesis contributes to phenotypic deficits in a 22q11-deletion mouse model. Nat Genet..

[34] Dolcetti A, Silversides CK, Marshall CR, Lionel AC, Stavropoulos DJ, Scherer SW (2013). 1q21.1 Microduplication expression in adults. Genet Med.

[35] Lowther C, Speevak M, Armour CM, Goh ES, Graham GE, Li C (2017). Molecular characterization of NRXN1 deletions from 19,263 clinical microarray cases identifies exons important for neurodevelopmental disease expression. Genet Med..

[36] Costain G, Lionel AC, Fu F, Stavropoulos DJ, Gazzellone MJ, Marshall CR (2014). Adult neuropsychiatric expression and familial segregation of 2q13 duplications. Am J Med Genet B Neuropsychiatr Genet..

[37] Hladilkova E, Baroy T, Fannemel M, Vallova V, Misceo D, Bryn V (2015). A recurrent deletion on chromosome 2q13 is associated with developmental delay and mild facial dysmorphisms. Mol Cytogenet..

[38] Isles AR, Ingason A, Lowther C, Walters J, Gawlick M, Stober G (2016). Parental origin of interstitial duplications at 15q11.2-q13.3 in schizophrenia and neurodevelopmental disorders. PLoS Genet..

[39] Hippolyte L, Maillard AM, Rodriguez-Herreros B, Pain A, Martin-Brevet S, Ferrari C (2016). The number of genomic copies at the 16p11.2 locus modulates language, verbal memory, and inhibition. Biol Psychiatry.

[40] Fung WL, Butcher NJ, Costain G, Andrade DM, Boot E, Chow EW (2015). Practical guidelines for managing adults with 22q11.2 deletion syndrome. Genet Med.

[41] Giorda R, Bonaglia MC, Beri S, Fichera M, Novara F, Magini P (2009). Complex segmental duplications mediate a recurrent dup(X)(p11.22-p11.23) associated with mental retardation, speech delay, and EEG anomalies in males and females. Am J Hum Genet.

[42] Mandrile G, Dubois A, Hoffman JD, Uliana V, Di Maria E, Malacarne M (2013). 3q26.33-3q27.2 microdeletion: a new microdeletion syndrome?. Eur J Med Genet.

[43] Peddibhotla S, Nagamani SC, Erez A, Hunter JV, Holder JL, Carlin ME (2015). Delineation of candidate genes responsible for structural brain abnormalities in patients with terminal deletions of chromosome 6q27. Eur J Hum Genet..

[44] Mitter D, Ullmann R, Muradyan A, Klein-Hitpass L, Kanber D, Ounap K (2011). Genotype-phenotype correlations in patients with retinoblastoma and interstitial 13q deletions. Eur J Hum Genet..

[45] Margari L, Colonna A, Craig F, Gentile M, Giannella G, Lamanna AL (2014). Microphthalmia with linear skin defects (MLS) associated with Autism Spectrum Disorder (ASD) in a patient with familial 12.9Mb terminal Xp deletion. BMC Pediatr.

[46] Genovese G, Fromer M, Stahl EA, Ruderfer DM, Chambert K, Landen M (2016). Increased burden of ultra-rare protein-altering variants among 4,877 individuals with schizophrenia. Nat Neurosci..

[47] Oguro-Ando A, Zuko A, Kleijer KT, Burbach JP (2017). A current view on contactin-4, -5, and -6: Implications in neurodevelopmental disorders. Mol Cell Neurosci..

[48] Akarsu S, Torun D, Bolu A, Erdem M, Kozan S, Ak M (2014). Mitochondrial complex I and III gene mRNA levels in schizophrenia, and their relationship with clinical features. J Mol Psychiatry..

[49] Tam GW, van de Lagemaat LN, Redon R, Strathdee KE, Croning MD, Malloy MP (2010). Confirmed rare copy number variants implicate novel genes in schizophrenia. Biochem Soc Trans..

[50] Curtis D, Consortium UK (2016). Practical experience of the application of a weighted burden test to whole exome sequence data for obesity and schizophrenia. Ann Hum Genet..

[51] Ohtake H, Shimohata T, Terajima K, Kimura T, Jo R, Kaseda R (2004). Adult-onset leukoencephalopathy with vanishing white matter with a missense mutation in EIF2B5. Neurology..

[52] Scheffer IE, Mefford HC (2014). Epilepsy: Beyond the single nucleotide variant in epilepsy genetics. Nat Rev Neurol..

[53] Tammimies K, Marshall CR, Walker S, Kaur G, Thiruvahindrapuram B, Lionel AC (2015). Molecular diagnostic yield of chromosomal microarray analysis and whole-exome sequencing in children with autism spectrum disorder. JAMA..

[54] Wolfe K, Strydom A, Morrogh D, Carter J, Cutajar P, Eyeoyibo M (2016). Chromosomal microarray testing in adults with intellectual disability presenting with comorbid psychiatric disorders. Eur J Hum Genet..

[55] Rehm HL (2017). A new era in the interpretation of human genomic variation. Genet Med..

[56] Sanders SJ, He X, Willsey AJ, Ercan-Sencicek AG, Samocha KE, Cicek AE (2015). Insights into autism spectrum disorder genomic architecture and biology from 71 risk loci. Neuron..

[57] Borlot F, Regan BM, Bassett AS, Stavropoulos D, Andrade DM (2017). Prevalence of pathogenic copy number variation in adults with epilepsy and intellectual disability. JAMA Neurology..

[58] Romans SM, Stefanatos G, Roeltgen DP, Kushner H, Ross JL (1998). Transition to young adulthood in Ullrich-Turner syndrome: neurodevelopmental changes. Am J Med Genet..

[59] Udwin O, Yule W (1991). A cognitive and behavioural phenotype in Williams syndrome. J Clin Exp Neuropsychol..

[60] Bearden CE, Woodin MF, Wang PP, Moss E, McDonald-McGinn D, Zackai E (2001). The neurocognitive phenotype of the 22q11.2 deletion syndrome: selective deficit in visual-spatial memory. J Clin Exp Neuropsychol.

[61] Gerber S, Alzayady KJ, Burglen L, Bremond-Gignac D, Marchesin V, Roche O (2016). Recessive and dominant de novo ITPR1 mutations cause Gillespie syndrome. Am J Hum Genet..

[62] van de Leemput J, Chandran J, Knight MA, Holtzclaw LA, Scholz S, Cookson MR (2007). Deletion at ITPR1 underlies ataxia in mice and spinocerebellar ataxia 15 in humans. PLoS Genet..

[63] Huang L, Chardon JW, Carter MT, Friend KL, Dudding TE, Schwartzentruber J (2012). Missense mutations in ITPR1 cause autosomal dominant congenital nonprogressive spinocerebellar ataxia. Orphanet J Rare Dis..

[64] Trikamji B, Singh P, Mishra S (2015). Spinocerebellar ataxia-10 with paranoid schizophrenia. Ann Indian Acad Neurol..

[65] Rottnek M, Riggio S, Byne W, Sano M, Margolis RL, Walker RH (2008). Schizophrenia in a patient with spinocerebellar ataxia 2: coincidence of two disorders or a neurodegenerative disease presenting with psychosis?. Am J Psychiatry..

[66] Joo EJ, Lee JH, Cannon TD, Price RA (1999). Possible association between schizophrenia and a CAG repeat polymorphism in the spinocerebellar ataxia type 1 (SCA1) gene on human chromosome 6p23. Psychiatr Genet..

[67] Seidman LJ, Buka SL, Goldstein JM, Tsuang MT (2007). Intellectual decline in schizophrenia: evidence from a prospective birth cohort 28 year follow-up study. J Clin Exp Neuropsychol..

