# Supplementary material for: Impact of IQ on the diagnostic yield of chromosomal microarray in a community sample of adults with schizophrenia
Source: Genome Med. 2017 Nov 30;9:105. doi: 10.1186/s13073-017-0488-z (PMC5708103; doi:10.1186/s13073-017-0488-z)
Supplement: Supplementary file 1 — A word document that contains one figure that depicts the verbal and performance IQ scores for 29 individuals with schizophrenia and a NVLD (Figure S1), and three tables, including: (1) a list of 10,113 population-based controls used to adjudicate CNV rarity in schizophrenia participants (Table S1); (2) the demographic and clinical information for 546 probands with schizophrenia of European ancestry (Table S2); and (3) the genome-wide burden of all rare autosomal CNVs > 10 kb between the expanded schizophrenia-LIQ and schizophrenia-average IQ groups (Table S3). (DOCX 87 kb) [file 13073_2017_488_MOESM1_ESM.docx]

**ADDITIONAL FILE 1**

**Impact of IQ on diagnostic yield of chromosomal microarray in a community sample of adults with schizophrenia**

Chelsea Lowther^1,2^, Daniele Merico^3,4^, Gregory Costain^1,5^, Jack Waserman^6^, Kerry Boyd^7^, Abdul Noor^8^, Marsha Speevak^8^, Dimitri J. Stavropoulos^8^, John Wei^4^, Anath C. Lionel^4^, Christian R. Marshall^4,8,9^, Stephen W. Scherer^4,10^, Anne S. Bassett^1,2,11^

| **Document** | **Page** |
| --- | --- |
| Additional file 1: Figure S1. Verbal and performance IQ scores for 29 subjects with schizophrenia and a non-verbal learning disability | 2 |
| Additional file 1: Table S1. List of 10,113 population-based controls used to adjudicate CNV rarity in schizophrenia subjects | 3 |
| Additional file 1: Table S2. Demographic and clinical information for 546 probands with schizophrenia of European ancestry | 4-5 |
| Additional file 1: Table S3. Genome-wide burden of all rare autosomal CNVs > 10kb between the expanded schizophrenia-LIQ and schizophrenia-Average IQ groups | 6 |
| References | 7-8 |

**Additional file 1: Figure S1. Verbal and performance IQ scores for 29 subjects with schizophrenia and a non-verbal learning disability**

**
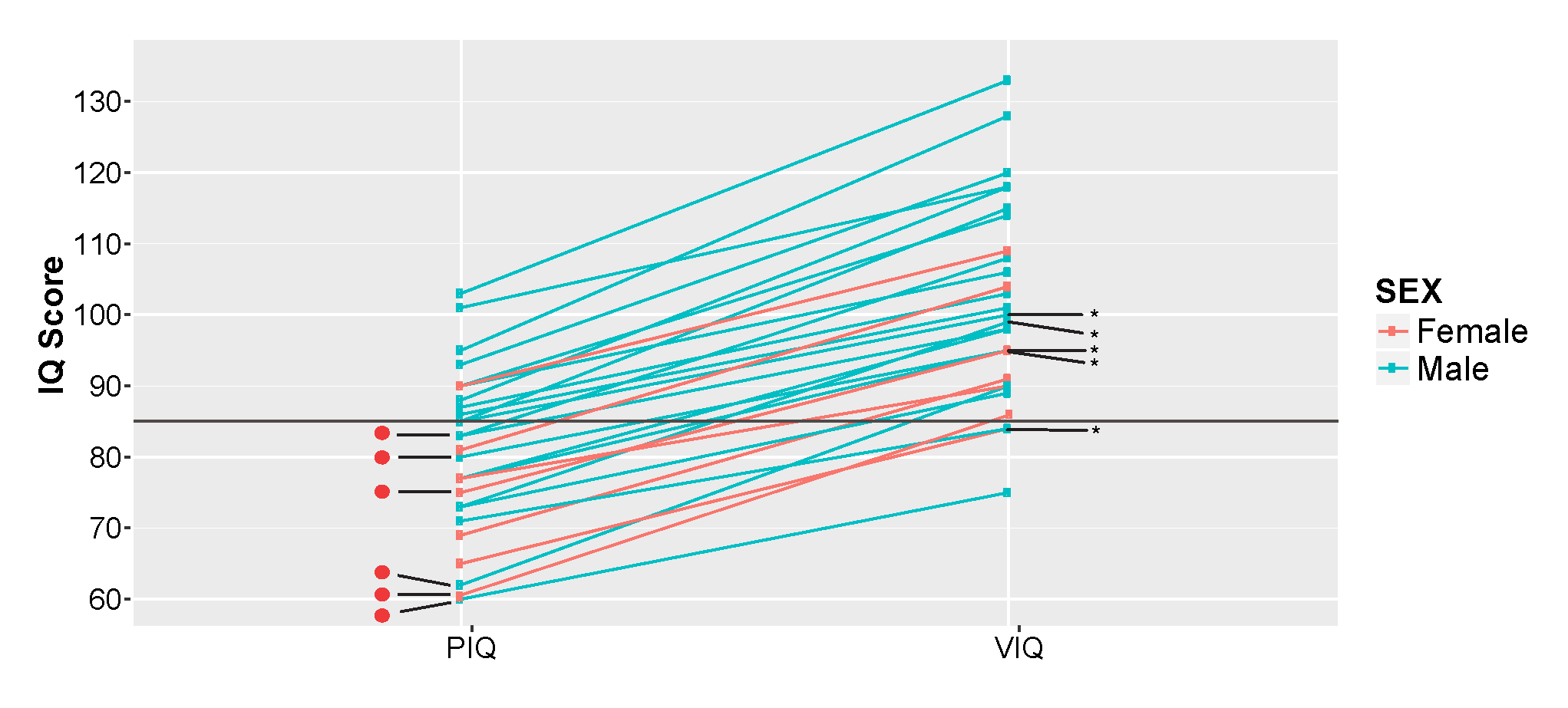
**

Raw IQ scores were available for 24 of the 29 subjects with schizophrenia and a non-verbal learning disability (schizophrenia-NVLD; defined as having a performance IQ [PIQ] score ≥15 points lower than their verbal IQ score [VIQ]).[1] For the five schizophrenia-NVLD individuals with only descriptive IQ ranges available (denoted by an asterisk in the figure), we used the median PIQ and VIQ scores for that particular range (e.g., average, borderline, mild, etc.) based on population norms.[2, 3] We have annotated the six subjects that were found to have a pathogenic CNV using a red circle. The dark grey horizontal line denotes an IQ score of 85, which was used as the cut off for assigning individuals to the schizophrenia-LIQ or average IQ groups in the rest of the sample. All but one of the six subjects with a pathogenic CNV had a VIQ >85. There were significantly (p=0.016) more males (n=22; 75.9%) than females (n=7; 24.1%) with schizophrenia and a NVLD, but this ratio was comparable to that for the overall sample of n=546 subjects (67.9% male, 32.1% female; Additional file 4). VIQ, verbal IQ; PIQ, performance IQ.

**Additional file 1: Table S1. List of 10,113 population-based controls used to adjudicate CNV rarity in schizophrenia subjects**

| **Control datasets** | **Number of subjects** | **Array platform** | **Description of dataset** |
| --- | --- | --- | --- |
| Ottawa Heart Institute (OHI) controls | 1,234 | Affymetrix 6.0 | Stewart et al. 2009[4] |
| POPGEN | 1,123 | Affymetrix 6.0 | Krawczak et al. 2006[5] |
| Health, Aging, and Body Composition (Health ABC) study controls | 2,566 | Illumina 1M Duo | Coviello et al. 2012[6] |
| SAGE consortium controls | 1,769 | Illumina 1 M | Bierut et al. 2010[7] |
| ONC (Ontario Familial Colorectal Cancer Registry) | 433 | Illumina 1 M | Cotterchio et al. 2005[8] |
| Collaborative Genetic Study of Nicotine Dependence (COGEND) controls | 1,213 | Illumina OMNI 2.5M Quad | Bierut et al. 2007[9] |
| KORA controls | 1,775 | Illumina OMNI 2.5M Quad | Verhoeven et al. 2013[10] |
| **TOTAL** | **10,113** | **-** |  |

**Additional file 1: Table S2. Demographic and clinical information for 546 probands with schizophrenia of European ancestry**

|  |  | **Schizophrenia-LIQ group (n=192)** | |  |  |
| --- | --- | --- | --- | --- | --- |
|  | **Total sample (n=546)** | **Mild-mod ID**  **(n=62 [11.4 %])** | **Borderline IQ**  **(n=130 [23.8%])** | **Average IQ**  **(n=325 [59.5%])** | **NVLD**  **(n=29 [5.3%])** |
| **Current age** |  |  |  |  |  |
| Male, mean (SD) | 52.9 (12.5) ** | 53.0 (12.1) | 53.0 (11.6) | 52.6 (12.9) ** | 56.1 (13.0) |
| Female, mean (SD) | 56.6 (12.6) | 55.6 (13.0) | 56.0 (13.6) | 57.3 (12.0) | 57.3 (12.0) |
| **Sex** |  |  |  |  |  |
| Male, N (%) | 371 (67.9) | 33 (53.2) | 83 (63.8) | 233 (71.7) | 22 (75.8) |
| Female, N (%) | 175 (32.1) *** | 29 (46.8) | 47 (36.2) * | 92 (28.3) *** | 7 (24.2) *** |
| **AAO**^a^ |  |  |  |  |  |
| Male, mean (SD) | 22.1 (6.0) * | 23.8 (10.7) | 22.4 (5.9) | 22.0 (5.2) *** | 20.5 (3.9) |
| Female, mean (SD) | 24.5 (8.9) | 22.7 (8.0) | 22.4 (7.4) | 26.3 (9.6) | 21.7 (7.2) |
| **FSIQ**, N (%) | 136 (24.9) | 39 (62.9) | 45 (34.6) | 48 (14.8) | NA^b^ |
| Male, mean (SD) | 84.1 (16.4) | 63.7 (6.0) | 77.3 (3.9) | 98.9 (10.7) |  |
| Female, mean (SD) | 72.1 (14.2) *** | 58.9 (6.7) * | 75.1 (4.7) | 93.0 (5.9) * |  |
| **Years of education**    Male, mean (SD)  Female, mean (SD) | 11.7 (2.6)  11.7 (2.6)  11.7 (2.7) | NA^c^ | 9.4 (2.4)  9.1 (2.4)  10.0 (2.4) | 12.5 (2.2)  12.5 (2.1)  12.6 (2.4) | 12.1 (2.5)  12.2 (2.3)  12.2 (3.1) |
| **Previous clinical genetic testing**^d^,  N (%) | 7 (1.3) | 6 (9.7) | 1 (0.8) | 0 | 0 |

We performed comparisons between males and females within each IQ sub-group for each demographic variable. For continuous variables (age, age at onset, FSIQ) we used a two-tailed Student’s t-test and for the sex categorical variable we used a Chi-square test. Statistically significant results are indicated by asterisks: * = p<0.05, ** = p<0.01, and *** = p< 0.001. Non-significant differences between sexes are not shown. Mod, moderate; ID, intellectual disability; LIQ, low IQ; NVLD, non-verbal learning disorder; N, number of cases with demographic data available; %, percentage; SD, standard deviation; AAO, age at onset; FSIQ, full scale IQ. ^a^Age at onset for schizophrenia was defined as the first time the patient received treatment (as an outpatient or at inpatient hospitalization) for psychotic symptoms. ^b^Full scale IQs for individuals with a non-verbal learning disability are not reported because the discrepancy between verbal IQ and performance IQ makes this measure inaccurate.[1] ^c^Years of education for individuals with schizophrenia and ID are not provided because the majority of subjects were enrolled in special education or had modified curriculums. ^d^Detailed review of lifetime medical records was performed in order to identify any previous clinical genetic testing results for each participant, e.g., karyotype, fragile X testing, microarray, or fluorescence-in-situ hybridization.

**Additional file 1: Table S3. Genome-wide burden of autosomal CNVs > 10kb between the expanded LIQ and average IQ schizophrenia subgroups**

|  | **Schizophrenia-LIQ (n=191)^a^**  median; range | **Schizophrenia-average IQ (n=271)^a^**  median; range | **LIQ vs. Average IQ**  **OR (95% CI), p value^b^** |
| --- | --- | --- | --- |
| **Total number of rare autosomal CNVs** | | | |
| Total number of CNVs | 2; 1-13 | 2;1-8 | 1.14 (1.00-1.30), p=0.042 |
| Total number of deletions | 1; 0-11 | 1;0-5 | NS |
| Total number of duplications | 1; 0-6 | 1;0-5 | NS |
| **Total number of rare genic autosomal CNVs** | | | |
| Total number of genic CNVs | 1; 0-5 | 1; 0-6 | 1.19 (1.01-1.41), p=0.041 |
| Total number of genic deletions | 1; 0-3 | 1; 0-5 | NS |
| Total number of genic duplications | 1; 0-4 | 0; 0-3 | 1.42 (1.14-1.81), p=0.002 |
| **Genomic length of all rare autosomal CNVs (Mb)** | | | |
| Genomic length of all CNVs | 0.183; 0.01- 4.6 | 0.134; 0-3.97 | NS* |
| Genomic length of deletions | 0.053; 0-3.19 | 0.049; 0-2.70 | NS |
| Genomic length of duplications | 0.053; 0-4.6 | 0.037; 0-3.94 | NS |
| **Genic content of all rare autosomal CNVs (total number of genes overlapped)** | | | |
| Genic content of all CNVs | 2; 0-36 | 1; 0-33 | NS* |
| Genic content of deletions | 1; 0-21 | 0; 0-8 | NS |
| Genic content of duplications | 1; 0-35 | 0; 0-30 | NS |

LIQ, low IQ; OR, odds ratio; CI, confidence interval; NS, non-significant, p<0.05; *, p value at the trend level (p<0.1) ^a^The total number of individuals with an autosomal rare CNV after excluding n=39 subjects (n=24 with schizophrenia-LIQ, n=9 with schizophrenia-average IQ, and n=6 with a NVLD) with a pathogenic CNV. The remaining 23 subjects with schizophrenia-NVLD and no pathogenic CNV were added to an expanded schizophrenia-LIQ group. ^b^The p value was calculated using a logistic regression analysis, controlling for sex and genotyping platform.

**REFERENCES**

1. Harnadek MC, Rourke BP. Principal identifying features of the syndrome of nonverbal learning disabilities in children. J Learn Disabil. 1994;27:144-54.

2. Wechsler D: Measurement and appraisal of adult intelligence 4th edn. Baltimore, MD: Williams & Wilkens; 1958.

3. Kaufman AS, Kaufman JC, Liu X, Johnson CK. How do educational attainment and gender relate to fluid intelligence, crystallized intelligence, and academic skills at ages 22-90 years? Arch Clin Neuropsychol. 2009;24:153-63.

4. Stewart AF, Dandona S, Chen L, Assogba O, Belanger M, Ewart G, et al. Kinesin family member 6 variant Trp719Arg does not associate with angiographically defined coronary artery disease in the Ottawa Heart Genomics Study. J Am Coll Cardiol. 2009;53:1471-2.

5. Krawczak M, Nikolaus S, von Eberstein H, Croucher PJ, El Mokhtari NE, Schreiber S. PopGen: population-based recruitment of patients and controls for the analysis of complex genotype-phenotype relationships. Community Genet. 2006;9:55-61.

6. Coviello AD, Haring R, Wellons M, Vaidya D, Lehtimaki T, Keildson S, et al. A genome-wide association meta-analysis of circulating sex hormone-binding globulin reveals multiple Loci implicated in sex steroid hormone regulation. PLoS Genet. 2012;8:e1002805.

7. Bierut LJ, Agrawal A, Bucholz KK, Doheny KF, Laurie C, Pugh E, et al. A genome-wide association study of alcohol dependence. Proc Natl Acad Sci U S A. 2010;107:5082-7.

8. Cotterchio M, Manno M, Klar N, McLaughlin J, Gallinger S. Colorectal screening is associated with reduced colorectal cancer risk: a case-control study within the population-based Ontario Familial Colorectal Cancer Registry. Cancer Causes Control. 2005;16:865-75.

9. Bierut LJ, Madden PA, Breslau N, Johnson EO, Hatsukami D, Pomerleau OF, et al. Novel genes identified in a high-density genome wide association study for nicotine dependence. Hum Mol Genet. 2007;16:24-35.

10. Verhoeven VJ, Hysi PG, Wojciechowski R, Fan Q, Guggenheim JA, Hohn R, et al. Genome-wide meta-analyses of multiancestry cohorts identify multiple new susceptibility loci for refractive error and myopia. Nat Genet. 2013;45:314-8.
